# Predictors of COVID-19 Fatality: A Worldwide Analysis of the Pandemic over Time and in Latin America

**DOI:** 10.1007/s44197-022-00031-x

**Published:** 2022-01-14

**Authors:** Dayana Rojas, Jorge Saavedra, Mariya Petrova, Yue Pan, José Szapocznik

**Affiliations:** 1 Department of Public Health Sciences, Don Soffer Clinical Research Center, University of Miami, 1120 NW 14th Street, Miami, FL 33136-2107 USA; 2 Don Soffer Clinical Research Center, AHF Global Public Health Institute, 1120 NW 14th Street, Miami, FL 33136-2107 USA; 3 DeWitt Daughtry Family Department of Surgery, University of Miami, Biomedical Research Building, 1501 NW 10th Avenue, Miami, FL 33136-1012 USA

**Keywords:** SARS-CoV-2 testing, COVID-19 mortality, Latin America, Policy

## Abstract

SARS-CoV-2 has infected over one hundred million people worldwide and has affected Latin America particularly severely in terms of both cases and deaths. This study aims to determine the association between SARS-CoV-2 testing and COVID-19 fatality rate worldwide over 8 months and to examine how this relationship differs between Latin America and all other countries. This cross-sectional study used March 2021 data from 169 countries. Multivariate regressions predicted COVID-19 fatality (outcome) from the number of SARS-CoV-2 tests (exposure), while controlling for other predictors. Results for March 2021 were compared to results from June 2020. Additionally, results for Latin America were also compared to all other countries except Latin American for March 2021. SARS-CoV-2 testing was associated with a significant decrease in COVID-19 fatality rate in both June 2020 and March 2021 (RR = 0.92; 95% CI 0.87–0.96 and RR = 0.86; 95% CI 0.74–1.00, respectively). SARS-CoV-2 testing was associated with a significant decrease in COVID-19 fatality rate in Latin American countries but not in all other countries (RR = 0.45; 95% CI 0.23–0.89 and RR = 0.95; 95% CI 0.82–1.11, respectively). However, the difference between the risk ratios for June 2020 and March 2021 and between the risk ratios for Latin America and all other countries were not statistically significant. Increased SARS-CoV-2 testing may be a significant predictor of lower COVID-19 case fatality rate, specifically in Latin American countries, due to the existence of a strong association, which may have driven the worldwide results.

## Introduction

As of March 2021, there have been over 120 million cases and over 2.5 million deaths attributed to COVID-19 worldwide [1]. Over 44% of all cases and 48% of all deaths were found in the Americas, primarily in the United States, Brazil, Colombia, Argentina, and Mexico [1]. While the United States has the highest number and percent per population of cases and deaths in the Americas, they also have abundant resources dedicated to limiting the spread of disease, treating cases, and vaccinating their population. In contrast, Latin American countries, which have the second, third, and fourth highest numbers of cases, and an overall larger number of deaths, have limited resources to address the pandemic. As of March 2021, roughly 19% of all COVID-19 cases and 28.5% of all COVID-19 deaths worldwide were attributed to Latin America, while the region accounts for approximately 8.4% of the total world population [1, 2]. Thus, Latin America has more than a two-fold over-representation of cases as compared to the size of its population [3–5].

Liang et al. published in *Nature Magazine* in June 2020, explored factors associated with variation in fatality rates worldwide [6]. The results of their cross-sectional analysis of 169 countries indicated that COVID-19 fatality rate decreased with increases in the number of tests per 100 people, government effectiveness, and number of hospital beds; and increased with increased proportions of the population aged 65 or older and quality of transportation infrastructure. Since this paper was published, there have been many changes associated with the management and response to the pandemic, including the evolution of testing and treatment policies and practices across the globe. At the time of the Liang et al*.* study, most countries were implementing strict testing policies, where only those individuals with symptoms who met specific criteria were tested [6, 7]. However, this is no longer the case due to an increase in testing resources in most countries.

Since the publishing of the Liang study, and the data analysis for this current study, there have been other studies with coinciding results suggesting that higher testing frequency has the potential to significantly lower COVID-19 case-fatality rate and prevent a significant number of deaths [8–14]. However, none of these studies specifically focus on the worldwide association between testing and COVID-19 fatality or the association in all Latin American countries, which is why we chose to focus on the Liang study and use their methods as a foundation for our study and a point of comparison to study this association as the pandemic evolved.

The main purpose of this study was to explore the effect of worldwide COVID-19 testing on fatality at two time-points during the pandemic, June 2020 and March 2021. Because in March 2021, at the time the second data draw was done, nearly a fifth of all COVID-19 cases and a fourth of COVID-19 deaths were in Latin America, we were also interested in exploring the predictors associated with deaths in Latin America, as compared to the rest of the world. Therefore, we established three aims. AIM 1 was to determine the association between COVID-19 testing and fatality, using worldwide COVID-19 data as of March 2021. AIM 2 was to determine whether the association between COVID-19 testing and fatality changed from June 2020 to March 2021. AIM 3 was to examine the differences in the role of testing in predicting COVID-19 fatality between Latin American and the rest of the world.

## Methods

### Study Design and Data Sources

Consistent with Liang et al*.* which was used as a point of comparison to gauge the progression of the association over time, we included data for 169 countries in this cross-sectional, ecological study and utilized data from the same open access databases [6]. COVID-19-related data were obtained from Worldometer, a website which contains daily reports on cumulative COVID-19 case numbers, deaths, critical cases and number of tests by country [15]. We obtained data for the present study on March 4th, 2021 at 12:00 PM EST.

Additionally, we gathered variables on non-COVID-19, country-related factors. Information regarding government effectiveness was obtained from the Worldwide Governance Indicators project website [16]. Proportion of people aged 65 or older, number of hospital beds, and communicable disease death rates were obtained from the World Development Indicators website [17]. Information on transport infrastructure quality was obtained from the Logistics Performance Index website [18]. The data for the country-related factors included in this analysis were downloaded for the latest years available.

### Variables

Consistent the World Health Organization, COVID-19 fatality rate was defined as the number of deaths from COVID-19 per 100 COVID-19 cases [19]. The other COVID-19 variables included in the analyses were number of tests per 100 people, number of COVID-19 cases per 1000 people, and COVID-19 critical case rate, defined as the number of critical cases per 100 COVID-19 cases. To improve model fit, we log-transformed two right-skewed COVID-19 variables: fatality rate and number of tests per 100 people.

Country-level variables included the proportion of people aged 65 or older, number of hospital beds per 1000 people, communicable disease death rate, transport infrastructure quality score (range 1–5, higher scores better), and government effectiveness score (range  – 2.5 to 2.5, higher scores better).

### Analyses

Aim 1. To determine the world-wide association between COVID-19 testing and fatality: first, we looked at the descriptive statistics for all variables; second, multivariate regression analyses were conducted to test the association between COVID-19 fatality and number of tests per 100 people, while controlling for case number per 1,000 people, critical case rate, government effectiveness, population aged 65 or older, hospital bed number per 1000 people, communicable disease death rate, and transport infrastructure quality. Country populations were used as weights to account for unequal variances. AIM 2. Comparing the analysis between the two time points allowed us to examine the extent to which testing affects COVID-19 fatality and how that relationship evolved from June 2020 to March 2021. For this aim, we compared the results obtained in AIM 1 to the results from the June 2020 study. Descriptive statistics were compared using Student’s *t* tests to calculate the differences between the COVID-19 related data at the two time-points, while the risk ratios that resulted from the regressions were compared using the ratio of risk ratios method [20]. Aim 3. Finally, using the same analytical approach as in Aim 1, the impact of testing on fatality rates for Latin America countries was compared to all other countries. The mean rate for the countries in each of the two groups were computed and the compared. Once more, Student’s *t* tests were used to compare the descriptive statistics between all-countries except Latin America and Latin American countries, while the regression risk ratios were compared using the ratio of risk ratios method [20]. Using the Encyclopedia Britannica’s definition of Latin America, we included the following Latin American countries in the study: Argentina, Belize, Bolivia, Brazil, Chile, Colombia, Costa Rica, Cuba, Dominican Republic, Ecuador, El Salvador, Guatemala, Haiti, Honduras, Mexico, Nicaragua, Panama, Paraguay, Peru, Uruguay, and Venezuela [21]. Analyses were performed using SAS 15.1

## Results

### Aim 1

#### Descriptive Statistics

As of March 2021, there were 115,757,888 cases of COVID-19 and 2,573,030 COVID-19-related deaths (Table 1). The mean COVID-19 fatality rate for the current study in March 2021 was 2.19% (95% CI 1.83–2.54). The mean number of tests per 100 people was 42.74 (95% CI 33.11–52.37); the mean number of cases per 1000 people was 25.65 (95% CI: 21.23 to 30.08); and the mean critical case rate was 0.18% (95% CI 0.09–0.27). The mean government effectiveness score was -0.01 (95% CI  – 0.17 to 0.16), the mean proportion of the population aged 65 and older was 9.17% (95% CI 8.15–10.18), the average bed number per 1000 people was 3.10 (95% CI 2.68–3.51), the mean communicable disease death rate was 21.95% (95% CI 18.68–25.23), and the mean transport infrastructure quality score was 2.76 (95% CI 2.65–2.87).

**Table 1:** Comparison of descriptive statistics of study variables—Aims 1 and 2

	a. Liang et al. (June 2020)^6^				b. Current study (March 2021)				Mean difference (95% CI)
	N	Mean	SE	95% CI	N	Mean	SE	95% CI	
COVID-19 fatality rate (%)	169	3.70	0.28	3.15–4.25	169	2.19	0.18	1.83–2.54	( – 1.51 ( – 1.98, -1.04)*
COVID-19-related factors									
Number of tests per 100 people	154	3.75	0.47	2.82–4.69	157	42.74	4.88	33.11–52.37	38.99 (29.31, 48.67)*
Case number per 1000 people	169	1.69	0.25	1.20–2.18	169	25.65	2.24	21.23–30.08	23.96 (19.51, 28.41)*
Critical case rate (%)	120	0.56	0.06	0.44–0.68	130	0.18	0.04	0.09–0.27	-0.38 (-0.52, -0.24)*
Country-related factors									
Government effectiveness score	167	-0.01	0.08	-0.17–0.16	167	-0.01	0.08	-0.17–0.16	
Population aged 65 or older (%)	162	9.17	0.51	8.15–10.18	162	9.17	0.51	8.15–10.18	
Bed number per 1000 people	146	3.14	0.22	2.72–3.57	149	3.10	0.21	2.68–3.51	
Communicable disease death rate (%)	159	31.04	1.79	27.50–34.58	159	21.95	1.66	18.68–25.23	
Transport infrastructure quality score	153	2.75	0.05	2.64–2.86	146	2.76	0.06	2.65–2.87	

Liang et al.^6^ (June 2020): total cases: 47,990,084, total deaths: 1,222,767

Current study (March 2021): total cases: 115,757,888 total deaths: 2,573,030

^*^Denotes significant findings

Description: This table shows the descriptive statistics of the variables included in the analysis from the previous study (**a**.) and the current study (**b**.). As well as a statistical comparison of the difference between the variables at the two time points

#### The Worldwide Association Between COVID-19 Testing and Fatality, March 2021

In March 2021, an increase of one test per 100 people resulted in a significant 14% decrease in the COVID-19 fatality rate (RR = 0.860; 95% CI 0.740–0.999) (Table 2b). An increase in the critical case rate resulted in a significant 86% increase in the COVID-19 fatality rate (RR = 1.864; 95% CI 1.017–3.418), while an increase of one case per 1,000 people did not have a significant effect. A 0.1 increase in government effectiveness score (RR = 0.924; 95% CI 0.892–0.957) and a 1% increase in communicable disease death rate (RR = 0.984; 95% CI 0.972–0.995) significantly reduced COVID-19 fatality rates by 8% and 2%, respectively (Tables 3 and 4). A 0.1 increase in transport infrastructure quality score (RR = 1.112; 95% CI 1.069–1.156) significantly increased the fatality rate by 11%, while proportion of the population aged 65 or older and bed number per 1000 people had no significant effects. There were 105 countries with the necessary information to include in the regression analyses; excluded countries are shown in Appendix Table 5.

**Table 2:** Multivariate regression predicting the association between COVID-19 testing and COVID-19 fatality rates—Aims 1 and 2

	a. Liang et al. (June 2020) ^6^			b. Current study (March 2021)			Ratio of RRs (95%CI)
	RR	SE	95% CI	RR	SE	95% CI	
Number of tests per 100 people	0.92	0.02	0.87–0.96*	0.860	0.07	0.740–0.999*	0.97 (0.84, 1.12)
Case number per 1000 people	1.03	0.04	0.95–1.10	0.999	0.00	0.994–1.006	0.99 (0.91, 1.07)
Critical case rate (%)	1.05	0.06	0.94–1.18	1.864	0.80	1.017–3.418*	1.28 (0.27, 6.18)
Government effectiveness score	0.96	0.02	0.92–0.99*	0.924	0.02	0.892–0.957*	0.98 (0.93, 1.04)
Population aged 65 or older (%)	1.12	0.02	1.07–1.17*	1.002	0.02	0.957–1.050	0.95 (0.90, 1.01)
Bed number per 1000 people	0.85	0.03	0.80–0.90*	0.981	0.03	0.919–1.048	1.06 (0.98, 1.16)
Communicable disease death rate (%)	0.99	0.01	0.98–1.00	0.984	0.01	0.972–0.995*	1.00 (0.97, 1.02)
Transport infrastructure quality score	1.08	0.03	1.03–1.14*	1.112	0.02	1.069–1.156*	1.01 (0.94, 1.09)

Liang et al.^6^ (June 2020): 101 countries were included in the regression analysis

Current study (March 2021): 105 countries were included in the regression analysis

^*^Significant findings

Description: This table shows the risk ratios resulting from the multivariate regressions from the previous study (**a**.) and the current study (**b**.). In addition, a statistical comparison of the difference between the risk ratios at the two time points

**Table 3:** Comparison of descriptive statistics of study variables between Latin American countries and all other countries (as of March 4th, 2021)—Aim 3

	a. All countries (except Latin America)				b. Latin American countries only				Mean difference (95% C.I.)
	N	Mean	SE	95% CI	N	Mean	SE	95% CI	
COVID-19 fatality rate (%)	149	2.11	0.20	1.72–2.50	20	2.78	0.42	1.89–3.66	0.67 ( – 0.45, 1.79)
COVID-19-related factors									
Number of tests per 100 people	138	46.42	5.46	35.62–57.22	19	16.05	3.06	9.63–22.47	( – 30.37 (( – 42.75, ( – 17.99)*
Case number per 1000 people	149	25.69	2.47	20.81–30.57	20	25.39	4.56	15.84–34.94	( – 0.30 (( – 14.03, 13.43)
Critical case rate (%)	112	0.19	0.05	0.09–0.29	18	0.12	0.02	0.08–0.15	( – 0.07 (( – 0.18, 0.04)
Country-related factors									
Government effectiveness score	147	0.04	0.09	( – 0.14 to 0.22	20	( – 0.33	0.15	( – 0.64 to 0.03	( – 0.37 (( – 0.73, ( – 0.01)*
Population aged 65 or older (%)	142	9.29	0.58	8.14–10.43	20	8.31	0.66	6.92–9.69	( – 0.98 (( – 2.74, 0.78)
Bed number per 1000 people	129	3.31	0.23	2.85–3.77	20	1.72	0.28	1.14–2.31	( – 1.58 (( – 2.32, ( – 0.86)*
Communicable disease death rate (%)	139	23.06	1.87	19.36–26.76	20	14.28	1.39	11.37–17.18	( – 8.78 (( – 13.40, ( – 4.16)*
Transport infrastructure quality score	127	2.80	0.06	2.68–2.92	19	2.50	0.08	2.33–2.68	( – 0.30 (( – 0.50, ( – 0.10)*

For all countries except Latin America; total cases: 94,181,027, total deaths: 1,887,111

Overall COVID-19 fatality rate for all countries except Latin America (deaths/cases):

(1,887,111 / 94,181,027) × 100 = 2.00

Overall COVID-19 cases per 1000 people for all countries except Latin America (deaths/cases):

(94,181,027/6,940,918,714) × 1000 = 13.57

For Latin American countries only^8^; total cases: 21,576,861, total deaths: 685,919

Overall COVID-19 fatality rate for Latin American countries (deaths/cases):

(685,919/ 21,576,861) × 100 = 3.18

Overall COVID-19 cases per 1000 people for all countries except Latin America (deaths/cases):

(21,576,861/645,846,430) × 1000 = 33.41

^*^Significant findings

Description: This table shows the descriptive statistics of the variables included in the analysis from all countries except Latin America (**a.**) and the Latin American countries only (**b.**). As well as a statistical comparison of the variables between the two groups

**Table 4:** Multivariate regression predicting the association between COVID-19 testing and COVID-19 fatality rates (as of March 4th, 2021)—Aim 3

	**a**. All countries (except Latin America)			**b**. Latin American countries only			
	RR	SE	95% CI	RR	SE	95% CI	Ratio of RRs (95% CI)
Number of tests per 100 people	0.95	0.08	0.82–1.11	0.449	0.23	0.226–0.891*	0.72 (0.45, 1.17)
Case number per 1000 people	1.00	0.00	0.99–1.01	1.015	0.02	0.983–1.048	1.00 (0.97, 1.04)
Critical case rate (%)	1.68	0.69	0.93–3.02	35.087	1350.89	0.461–2669.322	3.74 (0, 10,000,000)
Government effectiveness score	0.89	0.02	0.86–0.93*	1.063	0.04	0.995–1.135	1.08 (0.99, 1.18)
Population aged 65 or older (%)	1.01	0.02	0.96–1.06	1.064	0.21	0.764–1.481	1.02 (0.68, 1.54)
Bed number per 1000 people	0.99	0.03	0.93–1.05	0.776	0.22	0.502–1.199	0.90 (0.58, 1.39)
Communicable disease death rate (%)	0.99	0.01	0.98–1.00	1.037	0.04	0.957–1.123	1.02 (0.94, 1.11)
Transport infrastructure quality score	1.15	0.02	1.10–1.20*	0.998	0.08	0.861–1.158	0.94 (0.8, 1.11)

All countries except Latin America: 87 countries were included in the regression analysis

Latin American countries only: 18 countries were included in the regression analysis

^*^Significant findings

Description: This table shows the risk ratios resulting from the multivariate regressions for the all countries except Latin America analysis (**a.**) and the Latin American countries only analysis (**b.**) Also, statistical comparison of the risk ratios between the two groups is shown

**Table 5:** Countries excluded from multivariate regressions due to missing variables

Country	Missing information
Algeria	Number of tests per 100 people
Andorra	Population aged 65 or older, Hospital beds per 1000 people, Communicable disease death rate, Transport infrastructure quality score
Angola	Hospital beds per 1000 people
Antigua and Barbuda	Transport infrastructure quality score
Armenia	Critical case rate
Aruba	Hospital beds per 1000 people, Communicable disease death rate, Transport infrastructure quality score
Australia	Critical case rate
Azerbaijan	Critical case rate, transport infrastructure quality score
Bangladesh	Critical case rate
Barbados	Critical case rate, transport infrastructure quality score
Belarus	Critical case rate
Belize	Transport infrastructure quality score
Benin	Critical case rate
Bermuda	Critical case rate, population aged 65 or older, hospital beds per 1000 people, communicable disease death rate, transport infrastructure quality score
Bosnia and Herzegovina	Critical case rate
Botswana	Transport infrastructure quality score
Brunei	Critical case rate
Burkina Faso	Number of tests per 100 people, Critical case rate
Burundi	Critical case rate
Cabo Verde	Transport infrastructure quality score
Cayman Islands	Critical case rate, population aged 65 or older, hospital beds per 1000 people, Communicable disease death rate, Transport infrastructure quality score
Chad	Number of tests per 100 people, Critical case rate, hospital beds per 1000 people
Comoros	Number of tests per 100 people, Critical case rate
Congo	Critical case rate, hospital beds per 1000 people
Djibouti	Critical case rate
Democratic Republic of Congo	Number of tests per 100 people, critical case rate, hospital beds per 1000 people
Eswatini	Transport infrastructure quality score
Ethiopia	Transport infrastructure quality score
Georgia	Critical case rate
Guinea-Bissau	Hospital beds per 1000 people
Haiti	Critical case rate
Hong Kong	Hospital beds per 1000 people, Communicable disease death rate
Indonesia	Critical case rate
Ivory Coast	Critical case rate, hospital beds per 1000 people
Libya	Critical case rate
Liechtenstein	Population aged 65 or older, hospital beds per 1000 people, communicable disease death rate, transport infrastructure quality score
Maldives	Hospital beds per 1000 people
Mali	Critical case rate
Marshall Islands	COVID-19 fatality rate, number of tests per 100 people, critical case rate, population aged 65 or older, communicable disease death rate, transport infrastructure quality score
Mauritania	Hospital beds per 1000 people
Mauritius	Critical case rate
Monaco	Government effectiveness score, Population aged 65 or older, Communicable disease death rate, Transport infrastructure quality score
Mozambique	Critical case rate, transport infrastructure quality score
Myanmar	Critical case rate
Nepal	Critical case rate
New Zealand	Critical case rate
Nicaragua	Number of tests per 100 people, Critical case rate, transport infrastructure quality score
Nigeria	Hospital beds per 1000 people
North Macedonia	Transport infrastructure quality score
Palestine	Hospital beds per 1000 people, communicable disease death rate, transport infrastructure quality score
Rwanda	Hospital beds per 1000 people
San Marino	Government effectiveness score, Population aged 65 or older, communicable disease death rate, transport infrastructure quality score
Sao Tome and Principe	Critical case rate
Senegal	Hospital beds per 1000 people
Sierra Leone	Critical case rate, hospital beds per 1000 people
Somalia	Number of tests per 100 people, critical case rate
South Sudan	Hospital beds per 1000 people, transport infrastructure quality score
Sri Lanka	Critical case rate
Sudan	Number of tests per 100 people, critical case rate
Suriname	Transport infrastructure quality score
Syria	Number of tests per 100 people, critical case rate
Tajikistan	Number of tests per 100 people, critical case rate
Tanzania	Number of tests per 100 people, transport infrastructure quality score
Togo	Critical case rate
United Arab Emirates	Critical case rate

### Aim 2

#### Descriptive Statistics Comparing June 2020 and March 2021

There was a significant decrease in the COVID-19 fatality rate from June 2020 to March 2021 of 1.51% (95% CI  – 1.98,  – 1.04) (Table 1a and b). The results also show that the number of tests per 100 people and case number per 1000 people increased significantly over time (Mean Difference = 38.99; 95% CI 29.31–48.67 and Mean Difference = 23.96; 95% CI 19.51–28.41, respectively). In contrast, the critical case rate decreased significantly by 0.38% (95% CI  – 0.52 to  – 0.24). Country-level information was obtained from the same sources for the same time-points, and, thus, change over time was not explored.

#### The Worldwide Association Between COVID-19 Testing and Fatality for June 2020 and March 2021

The effect estimates for the COVID-19-related factors from March 2021 (Table 2b) to June 2020 (Table 2a) did not differ significantly. However, it is interesting to note that, in relation to fatality rates, bed number per 1000 people and proportion of the population aged 65 and older were significantly associated with fatality in June 2020, but the variables lost significance in March 2021 (RR = 0.85; 95% CI 0.80–0.90 and RR = 1.12; 95% CI 1.07–1.17 vs. RR = 0.981; 95% CI 0.919–1.048 and RR = 1.002; 95% CI 0.957–1.050, respectively). Additionally, communicable disease death rate was not significant in the previous study but was significant in the current study (RR = 0.99; CI 0.98–1.00 vs. RR = 0.984; CI 0.972–0.995).

### Aim 3

#### Descriptive Statistics

The Latin America analysis showed that the mean number of tests per 100 people was significantly smaller in Latin American countries (Table 3b) than in all other countries (Table 3a) (Mean Difference =  – 30.37; 95% C.I  – 42.75 to  – 17.99), which means that Latin America is testing almost three times less than all other countries. However, the mean COVID-19 fatality rate, mean case number per 1000 people, and mean critical case rate were not significantly different between the two groups (Table 3a and b). To determine the overall rates per total population for the region, we also computed overall fatality rate and overall cases per 1000 for Latin America and the rest of the world. The overall fatality rate was 3.18 for Latin America and 2.00 for the rest of the world and the overall cases per 1000 people was 33.41 for Latin America and 13.57 for the rest of the world.

Latin American countries have significantly lower perceptions of government effectiveness as compared to all other countries (mean difference =  – 0.37; 95% C.I.  – 0.73 to  – 0.01). Additionally, as compared to all other countries, Latin American countries had significantly less hospital beds per 1000 people (mean difference =  – 1.59; 95% CI  – 2.32 to  – 0.86); a significantly lower communicable disease death rate (mean difference =  – 8.78; 95% CI  – 13.4 to  – 4.16); and a significantly lower transport infrastructure quality score (mean difference =  – 0.30; 95% CI  – 0.50 to  – 0.10). There were no significant differences in the proportion of people aged 65 and older.

#### The Association Between COVID-19 Testing and Fatality in Latin America

An increase of one test per 100 people in all other countries (Table 4a) does not have a significant effect on fatality rate, whereas an increase of one test per 100 people in Latin America (Table 4b) significantly reduces COVID-19 fatality rate by a dramatic 55% (RR = 0.449; 95% CI 0.226–0.891); however, the difference in risk ratios between the two regressions was not statistically significant. Increases in case number per 1000 people, critical case rate, proportion of the population aged 65 and older, hospital beds per 1000 people, and communicable disease death rate did not have significant effects on fatality rates in either group and were not significantly different between the two groups. Government effectiveness score and transport infrastructure quality score did not have a significant effect on COVID-19 fatality rate in Latin America, but they were significant predictors of fatality in all other countries.

## Discussion

### Aim 1

In March 2021, increasing the number of tests per 100 people significantly reduced COVID-19 fatality rate worldwide, after adjusting for cases per 1000 people, critical case rate, government effectiveness score, percent of the population aged 65 and older, number of hospital beds per 1000 people, communicable disease death rate, and transport infrastructure quality score. Countries with greater government effectiveness scores had lower fatality rates, perhaps due to the availability of resources that produce effective interventions, policies, and procedures. Whereas communicable disease death rate had paradoxical results; if the communicable diseases death rate in a country is high, theoretically, then the COVID-19 fatality rate would also be high, instead of significantly lower. However, these results may be influenced by the COVID-19 anomaly in Africa, which, as of March 2021, had the highest communicable disease death rates and some of the lowest COVID-19 case and death rates in the world [19, 22]. Additionally, countries with better transport infrastructure quality scores had higher fatality rates, potentially due to ease of mobility in disease spreading.

### Aim 2

Results revealed a significant reduction in the COVID-19 fatality rate from June 2020 to March 2021. This reduction may have resulted from the changes in testing policies over time. In the beginning of the pandemic, testing was restrictive and mostly performed on fatally ill patients with severe symptoms. Whereas, eight months later, testing policies expanded to anyone who needed and/or wanted to get tested, which would include patients with few or no symptoms. However, this reduction in mortality may also have stemmed from an increase in skill and knowledge of new treatments for the most dangerous consequences of COVID-19 [22–24]. In addition, number of tests per 100 people and case number per 1,000 people significantly increased over this period, while critical case rate significantly decreased. This may have occurred for similar reasons as mentioned above; as more cases of COVID-19 with few or no symptoms were identified, the ratio of critical cases to all cases would have decreased.

Although the risk ratio of testing on fatality rate was smaller in March 2021, likely due to the increase in overall COVID-19 testing, the difference between June 2020 and March 2021 was not statistically significant. Identifying mostly critical cases may have had a greater impact on fatality rates in June 2020, whereas identifying mostly asymptomatic cases may have reduced the impact of testing on fatality rates in March 2021, likely due to the fact that these cases are silently responsible for significant transmission but often do not lead to severe disease [25, 26]. The risk ratios for case number per 1,000 people remained non-significant, while the effect of critical case rate on fatality rates gained significance in March 2021. One of the most notable changes over this period among the country-related factors on fatality rate was proportion of the population aged 65 and older: the risk ratio for the impact of the proportion of the population aged 65 and older was no longer statistically significant in March 2021. This change could be attributed to advancement in treatment strategies, increased cautiousness from this age group, as well as the broadening of testing practices, which evolved to include people of all ages. Additionally, according to CDC reports, the number of deaths among people aged 65 and older significantly decreased after June 2020 [28, 29].

### Aim 3

Results showed a significant difference in the average testing rate, which indicates that Latin America tests almost three times less than countries in other regions of the world. The average fatality rate was not significantly higher in Latin American countries, but when computing the mortality rate for the region (total deaths per population) Latin America had almost 2.5 times the COVID-19 mortality rate than the rest of the world. While the average number of cases per 1000 people appeared to be equal, when analyzing total cases per population, Latin America had almost three times the number of cases per 1000 people as the rest of the world. Hence, the method used to examine fatality rate and number of cases per 1000 (Table 1) among all countries in the world yielded a different set of results when comparing Latin America to the rest of the world, with Latin American having a very high burden of disease as compared to the rest of the world [27–29].

The relationship between number of tests per 100 people and fatality was significant for Latin America and non-significant for all other countries, suggesting that Latin America may have influenced the significant association seen in the worldwide regressions. It is thus surprising that the ratio of risk ratios for this association did not differ significantly between Latin America and the rest of the world. The lack of statistical significance may be due to the considerable heterogeneity in the association between testing and fatality across Latin American countries, some of which may be acting as outliers.

In the case of Latin America, there were no other significant predictors of fatality rate. However, government effectiveness score and transport infrastructure quality score did have significant effects on the association between testing and fatality rate in countries outside of Latin America; higher government effectiveness translates into a lower COVID-19 fatality rate, whereas higher transport infrastructure quality leads to an increase in fatality rate. Additionally, having a larger proportion of the population 65 and older did not significantly affect COVID-19 fatality rate in either group. As previously stated in Aim 2, there may be a number of reasons for this result, specifically in Latin America: the standard error associated with the proportion of the population aged 65 and older was large, showing that there are countries with very high and countries with very low proportions of people aged 65 and older, there are also higher rates of comorbidities found in Latin America that may have a stronger influence on fatality than age, and finally, the habits of the elder population changed drastically over the course of the pandemic [30].

### Limitations

In our attempts to replicate the methods of Liang et al. we encountered several discrepancies in the data [6]. Primarily, we were unable to exactly match the countries in our dataset to the dataset from Liang et al. both of which were obtained for 2018, the most recent available data [6]. Specifically, three countries, which previously did not have any COVID-19 testing data, provided this between the times Liang et al*.* accessed the data (June 2020) and the time we accessed the 2018 data (March 2021), and an additional ten countries provided data on critical cases [6]. However, because we did not have the original dataset used by Liang et al. we were unable to determine for which countries we now had this additional data [6]. Also, the data we obtained in March 2021 for the country-related factors did not wholly match Liang et al. who did not specify how these data were obtained and if there were any issues with the information [6]. In our case, for example, the dataset for hospital beds per 1,000 people contained almost no information (79% of the data was missing) for the 169 countries in 2018, therefore we used as 10-year average instead. Similarly, the latest data available for communicable disease death rate was for 2016, not 2018 as Liang et al. stated [6]. Finally, we found less data points in the 2018 dataset for transport infrastructure quality score than what was presented in Liang et al. [6]. It is unclear how the data were downloaded from the same sources for 2018, the latest available year varied, but it could be a contributing factor to the differences observed between the two studies.

It is also important to note that not all potential factors and confounders associated with COVID-19 fatality rate were studied. There could be other important factors that are contributing to the fatality rate of COVID-19 that require further research, such as comorbidities, especially obesity, hypertension, and diabetes, particularly in Latin America. Additionally, biases associated with ecological studies must also be considered such as migration between countries and the ecological fallacy, which states that these results at the country level may not be generalized to the individual.

## Conclusions

The negative association between testing and COVID-19 fatality rate, which was most notable in Latin America, is indicative of the importance of testing, as it provides patients with the opportunity to properly isolate, and seek timely and appropriate medical care, which would reduce their risk of death. The findings of this paper demonstrate the differential responses to this public health crisis across specific countries and the implications for current and future testing policies and practices as a potential strategy for reducing fatality. Increasing resources, whether through government funding or international aid, targeted towards testing and treatments has the potential to substantially reduce the COVID-19 fatality rate, particularly in Latin America. Further research that is focused on testing and other COVID-19 regulation and management policies throughout specific countries is needed to fully understand the pandemic and determine ways to reduce COVID-19 fatality.

## Consent for Publication

Not applicable.

## Data Availability

The datasets analyzed during the current study are available via the following links: COVID-19-related data: Worldometers (https://www.worldometers.info/coronavirus/). Government effectiveness: Worldwide Governance Indicators project (https://info.worldbank.org/governance/wgi/). Proportion of people aged 65 or older, number of hospital beds, and communicable disease death rates: World Development Indicators (https://datacatalog.worldbank.org/dataset/world-development-indicators). Transport infrastructure quality score: Logistics Performance Index (https://lpi.worldbank.org/).

## Appendix

See Table 5.
